# Evaluation of Immunohistochemical Findings and Clinical Features Associated with Local Aggressiveness in Basal Cell Carcinoma

**DOI:** 10.30699/ijp.2019.82907.1781

**Published:** 2019-08-01

**Authors:** Simin ShamsiMeymandi, Shahriar Dabiri, Alireza ZeynadiniMeymand, Maryam Iranpour, Maryam Khalili, Sorour Alijani, Mahin Aflatoonian

**Affiliations:** 1 Pathology and Stem Cell Research Center, Dermatopathology Department, Afzalipour Teaching Hospital, Kerman University of Medical Sciences, Kerman, Iran; 2 Pathology and Stem Cell Research Center, Afzalipour Teaching Hospital, Kerman University of Medical Sciences, Kerman, Iran; 3 Department of Pathology, Afzalipour Teaching Hospital, Kerman University of Medical Sciences, Kerman, Iran; 4 Department of Dermatology, Dermatopathology Department, Afzalipour Teaching Hospital, Kerman University of Medical Sciences, Kerman, Iran

**Keywords:** Immunohistochemical, Aggressiveness, Basal Cell Carcinoma

## Abstract

**Background & Objective:**

Basal cell carcinoma (BCC) is classified into BCC1 or low risk (nodular, superficial type) and BCC2 or high risk (micronodular, morpheaform, infiltrative, and basosquamous types) based on clinical behavior. This study attempts to evaluate immunohistochemical (IHC) findings and clinical features associated with local aggressiveness and recurrence in BCC lesions.

**Methods:**

This is a cross-sectional descriptive study conducted on 42 paraffin blocks (22 BCC1, 20 in BCC2) at Pathology Department of Afzalipour Teaching Hospital. First, demographic features of the patients were recorded and pathology blocks were classified by two dermatopathologists based on histopathological types of BCC1 and BCC2. Then, primary monoclonal antibodies including CD10, CD1a, SMA, Ki67, CD34, and P53 were utilized for IHC study. We compared BCC1 and BCC2 according to IHC markers, demographic features of patients, and tumoral features.

**Results:**

The mean number of Langerhans cells (LCs) within epidermis above tumor mass was 14+1.92 and 4.7±1.23 in BCC1 and BCC2, respectively; these results show a significant difference between the two groups (*P*=0.001). P53 was positive in 41.13±6.39% and 74.5 ±6.26% of the tumor cells in BCC1 and BCC2 groups, which was statistically significant (*P*=0.001). Also, the mean number of blood vessels was 14.40±1.30 and 21.40±1.97 in BCC1 and BCC2, that was statistically significant (*P*=0.005).

**Conclusion:**

Higher numbers of angiogenesis (SMA positive) and positive P53 were observed in BCC2 than BCC1. Also, more active positive CD1a cells were observed in BCC1 compared to BCC2.

## Introduction

Basal cell carcinoma (BCC) is the most common skin neoplasm accounting for 75% of non-melanoma skin tumors. In addition to fair skin and chronic sun exposure which are the most predisposing factors in the pathogenesis of BCC, genetic syndromes (xeroderma pigmentosum, Rombo syndrome, Bazex syndrome), immune suppression, chemical exposure (arsenic, tar), and ionizing radiation contribute to its pathogenesis (1-3). Although metastasis is rarely seen secondary to BCC, local destruction and recurrences have been reported. Factors such as site and size of the lesion, histo-pathological subtypes, perineural and intravascular invasion are associated with recurrence and local aggressiveness of BCC lesions (4-7).

BCC is classified into BCC1 or low risk (nodular, superficial type) and BCC2 or high risk (micronodular, morpheaform, infiltrative, and basosquamous types) based on clinical behavior. BCC1 is characterized by cellular nests with peripheral palisading and cleft artifact. BCC2 is characterized by cellular nests infiltrated into collagenous stroma with high mitotic activity, necrotic cells, and deeper invasion within dermis and subcutaneous tissue without peripheral palisading and cleft artifact (8). This study, for the first time in Iran, attempted to evaluate immunehistochemical findings (IHC) and clinical features associated with local aggressiveness and recurrence in BCC lesions. 

## Materials and Methods

This is a cross-sectional descriptive study carried out on 42 paraffin blocks (22 BCC1, 20 in BCC2) at Pathology Department of Afzalipour Teaching Hospital. Demographic features of the patients including age, sex, location, and size of the lesions were recorded. Pathology blocks were revised by two pathologists with light microscope (Olympus, Bx53 series) and then classified into two groups of BCC1 and BCC2 based on their histopathological types. BCC1 included nodular and superficial types and BCC2 included micronodular, morpheaform, and basosquamous types.

For IHC study, histologic sections of paraffin blocks (3 mm thick) were deparaffinized with xylene and rehydrated with ethanol. Antigen retrieval was performed with Tris-EDTA (PH=9) buffer and microwaved for 10 minutes. Tissue peroxidase activity was deactivated by methanol 0.5% and H2O2. Then, primary monoclonal antibodies including CD10 (Dako 56C6, dilution factor (df) 1:30, Denmark), CD1a (Dako M 3571, df 1: 50, Denmark), SMA (Dako 1A4, df 1:1500, Denmark), Ki67 (Dako MIB1, df 1:50, Denmark) and P53 (Dako DO7, df 1:50, Denmark) were incubated for 30 minutes in room temperature. Secondary antibodies were added by biotin and streptavidin peroxidase technique. For demonstration of antigen-antibody bands, diaminobenzidine tetrahy-drochloride (DAB) chromogen was utilized and background was stained with hematoxylin.

To avoid bias, each slide was coded with a number and evaluated blindly by two pathologists. Counting of Langerhans cells (LCs) was done at 5 different fields of 0.32 mm^2 ^and the mean number of cells was recorded. Only LCs with obvious nucleus were counted in order to avoid recounting. For counting of blood vessels, firstly all the slides (stained with SMA) were observed by 10 high power fields (HPFs) to select fields with higher density of vessels. Then, counting was done by 200 HPFs in 3 fields; and the mean number, minimum and maximum of blood vessels were recorded. Only post-capillary venules, capillaries, and endothelial cells without lumen (single or clustered) were counted (8-10).

 For evaluation of SMA and CD10, modified quick score was used (7). Intensity and distribution of these markers were classified as follows: 0 (no staining), 1 (weak staining; only observed by high HPFs), 2 (moderate, easily observed by low HPFs), and 3 (strong; prominently observed by low HPFs).

Ki67 and P53 were recorded as percentage of positive staining cells among 100 tumoral cells. Finally, the 2 types of BCCs were compared according to IHC markers and demographic features of cases.

## Results

In 42 skin biopsy samples (22 BCC1 and 20 BCC2) were evaluated. The number of samples belonging to male patients was higher (52.4%) and the mean age of the patients was 67.69±12.29 (min=38, max=87) years old. Table (1) illustrates clinical features of BCC lesions.

The mean number of LCs within epidermis above tumor mass was 14+1.92 in BCC1 and 4.7±1.23 in BCC2, which indicated a significant difference between the two groups (*P*=0.001).

P53 was positive in 41.13±6.39% and 74.5 ±6.26% of the tumor cells in BCC1 and BCC2, respectively (statistically significant: *P*=0.001). Ki67 was positive in 28.54±28.54% and 45.8±7.74% in BCC1 and BCC2, respectively (*P*=0.081).

The mean number of blood vessels was 14.40±1.30 and 21.40±1.97 in BCC1 and BCC2 (statistically significant: *P*=0.005). SMA was positive in 72.7% and 90% in BCC1 and BCC2 groups, respectively (*P*=0.083). Meanwhile, CD10 was positive in 46.3% and 55% in BCC1 and BCC2, respectively (*P*=0.655) (Table2).

**Table1:** Clinical features of BCC lesions in 2 groups

Variables		BCC1	BCC2	Total	P-value
Site	High risk	10(45.5%)	9(45%)	19(45.2%)	0.976
	Low risk	12(54.5%)	11(55%)	23(54.8)	
Size	<3 cm	19(95%)	15(75%)	34(84%)	0.077
	>3cm	1(5%)	5(25%)	6(16%)	
Ulceration	Yes	10(45.5%)	16(80%)	26(61.9%)	0.021
	No	12(54.5%)	4(20%)	16(38.1%)	

**Table2:** Immunohistochemical results of SMA and CD10 in 2 groups of BCCs

Variables		BCC1	BCC2	P-Value
SMA	0	2(10%)	6(27.3%)	0.083
	1	7(35%)	12(54.5%)	
	2	7(35%)	2(9.1%)	
	3	4(20%)	2(9.1%)	
CD10	0	9(45%)	14(63.6%)	0.655
	1	6(30%)	5(22.7%)	
	2	2(10%)	1(4.5%)	
	3	3(15%)	2(19.1%)	

## Discussion

Our results revealed a significant difference between BCC1 and BCC2 regarding the mean number of LCs, blood vessels, and percentage of positivity for p53. Although positivity percentages of Ki67, SMA, and CD10 were higher in BCC2 compared to BCC1, the results were not statistically significant.

In the study by Esmaeili (2015), expressions of P53 and Ki67 were observed in 76% and 60% of BCC skin biopsies, respectively (vs. 57.02% and 36.76% in our study) (11). Also, there was no significant correlation between histopathological types of P53 and Ki67 expressions, but the expression of P53 was significantly different between the two groups in our study. Furthermore in our study similar to Esmaeili study, there was no significant correlation between age and P53 and Ki67.

Ki67 is associated with cell proliferation and can be seen with increased expression and intensity in tumors (12). P53 is responsible for regulation of cell cycle and it is essential for prevention of cancer development. P53 activates after DNA damage and repairs cell damages through induction of arrest in cell cycle or increasing cell apoptosis. Mutation in P53 can lead to failure in apoptosis and proliferation of damaged cells that have an essential role in cancer development. Factors including age, ultraviolet exposure, smoking, chemical exposure (arsenic, tar), and ionizing radiation can have an influence on the rate of P53 expression (13, 14). The discrepancy between the findings of our study and those of Esmaeili’s survey might be explained by difference in patient characterization and exposure to different environmental factors.

In a different study by Pilloni (2009), the expressions of P53, Ki67, and SMA were observed in 75%, 63% and 28.5% of the cases (vs. 57.02%, 36.76% and 80.9% in our study). The higher percentage of SMA positivity belonged to the lesions in the central of face (75%). Furthermore, SMA positivity was observed in 24% of high risk types of BCC and 50% of the ulcerated lesions. (15). In our study, SMA positivity was observed in 38.09% of the lesions located in central of face, 42.8% of high risk types of BCC, and 54.7% of ulcerated lesions. We evaluated SMA in tumor cells and adjacent stroma that was higher in BCC1 than BCC2, but did not observe any significant difference between them. SMA in normal tissue sparsely distributed in perivascular, perieccrine, and perifollicular structures. In addition, some previous studies in breast cancer demonstrated that actin can lead to secretion of metalloproteinase by myo-fibroblast, destruction of matrix, and higher mobility of tumor cells. Thus, higher percentage of SMA positivity is related to increased risk of local aggressiveness and metastasis (6, 7, 16).

Recent studies have also confirmed the association between tumor angiogenesis with local aggressiveness, rapid growth, and metastasis tumors such as melanoma, squamous cell carcinoma as well as breast, pulmonary, and prostate cancers. This could explain the importance of angiogenesis in nutrition of tumors (17). In our study, micro-vascular angiogenesis was observed more significantly in high risk BCC (21.40±1.97) than low risk BCC (14.40±1.30). Our results were compatible with those of Aslani and Staibano studies. In the study by Aslani conducted in Sari province of Iran, the mean number of blood vessels stained with VIII-related antigens were 50.24±6.72 and 20.9±5.56 in BCC2 and BCC1, respectively (8). This study revealed higher angiogenesis in BCC lesions with a size greater than 3 cm (14 cases) and ulceration (25 cases). This finding was compatible with our results.

In the present study, LCs were significantly higher in BCC1 (14±1.92) than BCC2 (4.7±1.23). Similarly, in the study by Santos (2010), the number of LCs was higher in BCC1 (6.70±2.68) than BCC2 (5.05±2.38) (5). Recent studies reported lower numbers of LCs in malignant tumors such as melanoma than normal skin and benign lesions such as actinic keratosis. This could be explained by essential role of LCs in stimulation of immune system for localization of tumor cells. Thus, the lower number of LCs is associated with increased risk of tumor aggressiveness and metastasis (18-20).

## Conclusion

In the present study, higher number of angiogenesis and positive P53 were observed in BCC2 than BCC1. Also, CD1a was significantly higher in BCC1 than BCC2. Although the positivity of Ki67, CD10, and SMA was higher in BCC1 than BCC2, the difference was not statistically significant.
